# Supramolecular Arrangement and Rheological Properties of Bisamide Gels

**DOI:** 10.1021/acs.langmuir.3c01100

**Published:** 2023-07-26

**Authors:** Elmira Ghanbari, Zian Chen, Pooja Padmanabhan, Stephen J. Picken, Jan H. van Esch

**Affiliations:** Advanced Soft Matter (ASM) Group, Chemical Engineering Department, Faculty of Applied Science (TNW), Delft University of Technology, 2629 HZ Delft, The Netherlands

## Abstract

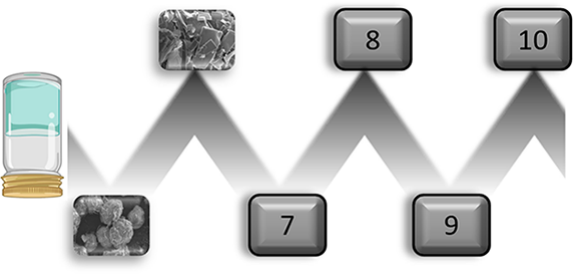

We report a systematic study of the gelation behavior of *n*BA gelators in xylene, with odd and even *n*-methylene spacers between the amide groups (*n* = 5–10) and 17 carbons at each end. The melting temperatures (*T*_m_^0^) of *n*BA gels are obtained from fitting our DSC_N_(T) model to the experimental DSC data. The found *T*_m_^0^ of *n*BA gels is about 35 °C lower than *T*_m_^0^ of the pure *n*BA gelators. This is reasonably well explained by a simple model combining theories of Flory–Huggins and Gibbs free energy of melting (FHM model). We attribute this depression to an increase in entropy upon melting of the gel due to mixing with the solvent. The odd–even alternation in *T*_m_^0^ of *n*BA gels, which was also found for the *n*BA gelators, indicates that the solid structures inside the gels are somewhat similar. This was studied using XRD: similar 00*l* reflections were found in the XRD patterns of all *n*BA gels and their *n*BA gelators. For even *n*BA gels, the same reflections in the 19–25° (2θ) region confirm that the sheetlike supramolecular structure of the gels is analogous to the lamellar structure of the solid gelators. For odd *n*BA gels, a slight difference in the reflections around 20–25° (2θ) implies a somewhat different side-by-side packing of odd *n*BA gels compared to the solid state. This variation is found for all the odd gels, and indeed, they show distinctly different morphologies compared to the even *n*BA gels. The possible effect of this on the rheological properties is discussed using some inspiration from the Halpin–Tsai model for composites where *n*BA gels are considered to be analogous to composite materials. The change of the storage modulus (*G*′) with the shape factor of woven fibers and sheets in *n*BA gels (20 wt %) indicates that a rheological odd–even effect might indeed be present.

## Introduction

Low molecular weight gelators (LMWGs) are small organic compounds (molecular weight ≤ 3000 Da)^1^ which can transform different types of organic liquids into solid-like viscoelastic gels.^2−7^ In recent years, such gel systems have found many potential applications in a variety of fields^8−11^ such as biomedicine,^12−18^ cosmetics, and food industry.^19−21^ LMWGs can form physical gels via specific noncovalent intermolecular interactions such as hydrogen bonding, van der Waals, π–π stacking, etc.^22−24^ and, hence, the gel formation is thermally reversible, which makes this type of gelators an excellent subject for studies into supra-molecular chemistry.^25^

Thermally triggered LMWG gels usually form via dissolution in a suitable solvent whereupon cooling sol-to-gel transition occurs.^26−28^ In fact, heating to elevated temperatures assists in overcoming intermolecular interaction forces among the gelator molecules in the crystalline solid state and dissolution of the gelators leading to sol formation. Upon cooling to below the gel transition temperature, molecules reassemble into a (new) crystalline state at a lower free energy.^29−31^ The assembly of gelator molecules into an interconnected solid-like three-dimensional (3D) network can immobilize the organic solvent which results in the formation of a strong or weak gel depending on the gelator–gelator interactions as well as the solvent properties.^32−34^

Due to a large structural diversity, gelators cannot be categorized into a single group representing their abilities to form gel. However, some features such as anisotropy in intermolecular interactions between the LMWGs are known as the driving force for their unidirectional assembly. As a result, they form fibers with large aspect ratios (length to cross-sectional diameter) which are able to efficiently immobilize a large quantity of solvent due to surface tension.^35,36^ A general requirement for a gel regardless of the solvent and gelator chemistry and their specific applications is the stability of the network on the time scale of observation, showing solid-like rheological properties.^32^

Production of a gel with desirable rheological properties for a certain application necessitates a good understanding of how rheological properties are governed by the microstructure and network properties.^37^ There is a need for a comparative study of molecular arrangement of gelators in the solid and supramolecular assembly of molecules in the gel state which can probe the link between the microscopic interactions between the constituent crystals of the gel network and the rheological properties of gels.^38,39^ Moreover, the formation of LMWG gels has been usually studied at relatively low mass fractions of LMWGs. A clear fundamental understanding of the physical properties of these gels at higher concentrations and how this impacts their rheological properties can provide essential information for potential applications and optimization.

**Figure 1:**
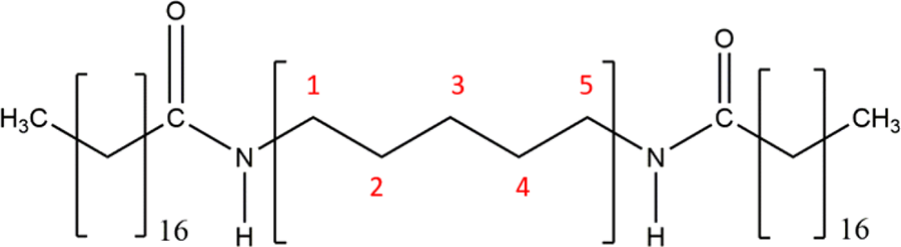
Chemical structure of synthesized bisamides (*n*BAs) with the (CH_2_)_*n*_ spacer between the amide groups (*n* = 5 in this example) and with C_17_ alkyl tails.

For this purpose, LMWGs that contain amide groups are among the best candidates to be studied. They have the most effective structural units for the formation of supramolecular gels due to their thermodynamically favored directional hydrogen bonding in a variety of solvents.^40^ Recently, in our research group, model bisamide gelators (*n*BAs) with the simplest structure were synthesized: linear aliphatic bisamides with two symmetric C17 alkyl tails, where the (CH_2_)_*n*_ spacer connecting the two amide groups was systematically varied from *n* = 5 to 10; see Figure 1. These were studied so as to determine their molecular arrangement and thermal properties in the solid state^41^ and to compare the corresponding gels state as we report here. Here, in this study, the effect of the spatial arrangement of amide groups on the supramolecular arrangement in the gel state and its influence on the microstructure and rheological properties is the topic of interest. The similarities and differences in molecular and supramolecular arrangement of the *n*BA gelators were investigated using calorimetry and X-ray diffraction. The differences in thermal properties of gels compared to the solid gelator state can be reasonably well explained by our Flory–Huggins melting (FHM) model, as described here. Finally, the implication of the odd-even effect on the microstructure and rheological properties of our gels is discussed using some inspiration from the Halpin–Tsai model.

## Materials and Methods

### Materials

Xylene ((CH_3_)_2_C_6_H_4_, analytical reagent grade, CAS:1330-20-7, purchased from Fisher Scientific) and a series of bisamide compounds (*n*BA) where *n* is the spacer length between amide groups (varying from 5 to 10) with 17 carbons at each side of the amide groups (C17) were used. The synthesis and characterization of the *n*BA gelators have been described in our previous research.^41^ The general chemical structure of *n*BA compounds is shown in Figure 1, using 5BA as an example:

### Gel Preparation

A certain amount of the synthesized *n*BA gelator was ground, weighed, and dispersed in the required weight of xylene in a vial. The mixture was used to prepare the *n*BA gel at different concentrations (5, 10, 20, 40 wt %) by mechanical stirring using a magnetic stir bar at 500 rpm and heating up to around 120 °C to dissolve the *n*BA gelator in the solvent using a heating block. Once the mixture became transparent, the vial was taken out and allowed to cool down to ambient temperature. A tube inversion test was conducted as a quick assessment of the gel formation immediately after cooling down and after 72 h.

### Differential Scanning Calorimetry

The thermal behavior of *n*BA gels was determined using a PerkinElmer-Pyris Diamond Differential scanning calorimeter with two 1(g)-furnaces (working on the Power-compensation temperature null principle.) Nitrogen (99.99% purity) was used to purge the thermal analysis system at the rate of 50 (mL/min). Temperature and heat flow calibration was done before each measurement using a heating scan of indium, a highly pure metal provided by PerkinElmer with accurately known enthalpy of fusion and melting point, Δ*H*_fusion_ = 28.47 J g^–1^ and *T*_m_^0^ = 156.4 °C, under the same condition as the to-be-measured samples. A *n*BA gel (8 ± 1 mg) was placed in a 40 μL stainless-steel sample pan by weighing on a microbalance. The sample pan and a reference pan (identical in terms of geometry and weight), both covered by stainless-steel lids, were placed in the furnaces of the DSC apparatus. Both the pans were heated to 130 °C, cooled to 25 °C, and subsequently followed the same heating cycle at a constant rate of 5 K min^–1^. The samples were kept isothermally for 2 min at the end of each scan.

### DSC_N_(T) Analytical Model and Curve Fitting

DSC_N_(T) function recently developed in our research group was fitted to the DSC traces of *n*BA gels.^41^
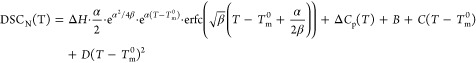
1

This model captures the shape of the experimental DSC peaks taking an assumed Arrhenius crystal size distribution, together with instrumental and sample-related Gaussian peak broadening, into account. Relying on DSC_N_(T), a much more accurate determination of the equilibrium melting point (*T*_m_^0^), enthalpy of fusion, and change in heat capacity of *n*BA gels has become possible. The nonlinear curve fitting of DSC_N_(T) to the experimental DSC traces was done using a Python 3 script^41^ and yields optimized values for the following parameters: Δ*H* (the coefficient of DSC_N_(T) function representing the change in enthalpy associated with the phase transition), *T*_m_^0^ (the equilibrium temperature of the phase transition), and α (the strength of the linearized Arrhenius function (α = *E*_a_/(*R*·(*T*_m_^0^)^2^) describing the crystal size distribution, roughly proportional to the steepness of the rising edge of the peak), β (the parameter in relation to the Gaussian broadening of the peak (, describing the peak broadening in the declining edge of the peak), and Δ*C*_p,m_ (the difference between the heat capacity of the pre- and post-transition states). The parameters *B*, *C*, and *D*, respectively, correct for baseline offset, linear baseline slope, and a second-order baseline curvature.

To assure the accuracy of the thermal properties obtained from DSC measurements, at least three *n*BA samples with a similar weight were measured under the same condition, and the data were analyzed after normalization per weight of the sample. The standard deviation of the thermal properties, melting temperature, enthalpy of fusion, heat capacity change, and the other fit parameters were obtained by fitting the analytical model DSC_N_(T) to the three sets of raw data which contain the experimental error along with the fitting procedure error. The experimental errors for the thermal properties of all *n*BA samples were less than 1%. The fitting deviation for each parameter was obtained from the residuals of nonlinear least squares (NLLS). The reported errors margins of the fit parameters in the table are the residuals of the NLLS rounded to two digits.

### X-ray Diffraction

X-ray diffraction (XRD) was used to obtain information on the crystal structure of *n*BA gels. XRD patterns were recorded at room temperature with a Bruker D8 Advance ECO diffractometer in Bragg–Brentano geometry, equipped with a Cu X-ray source (K_α1_ = 1.54060 Å and K_α2_ = 1.54439 Å) and a LYNXEYE-XE-T position sensitive detector. A knife edge has been used to reduce the background due to the scattering of the primary beam. The patterns were measured from 0.6° to 50°(2θ) with a step size of 0.01° and measuring time of 0.5 s per step. The intensity of reflections in counts s^–1^ was recorded which is then normalized with respect to the reflection with the highest intensity, the reflection of (001). This method of normalization enables us to compare the low-intensity reflections of the gels with their respective reflections of the gelators which have higher intensities.

### Scanning Electron Microscopy

The microstructure of *n*BA gels was observed by using a JEOL JSM 6010LA scanning electron microscope. A SEM sample was prepared by taking a small amount of a freshly made gel gently placed on aluminum foil covering the microscope slide. All samples were dried in a VT6025 vacuum oven (Thermo Electron Corporation) for 3 h at 60 °C. This temperature has been chosen safely below the melting point of the gel and its constituents to avoid any morphological change of the samples. Subsequently, the prepared sample was coated with gold particles at 20 mA for 30 s to increase conductivity for better image quality. The images were recorded using the following setting: secondary electron images (SEI) mode, 8 kV, WD10 mm, and SS40 at different magnifications (500×, 1000×, and 2500×). The dimensions of the fibers and sheets in the SEM images from the microstructures of the gels were measured. At least, ten SEM images were collected from different parts of the sample to assess the gel structure and their dimensions more accurately.

### Rheology

The rheological properties of the *n*BA gels were assessed by DISCOVERY HR-3 hybrid rheometer (TA Instrument). The used geometry was 40 mm parallel steel plate Peltier plate steel-999580. The temperature was set at 25 °C and the gap was set to 500 μm. The inertia, friction, and rotational mapping were calibrated. Zero gap was determined after the calibration of the geometry. Around 1.0 ± 0.2 g of a freshly prepared *n*BA gel sample was placed evenly on the bottom plate. The upper parallel plate was lowered to the geometry gap. To avoid solvent evaporation during the measurement, a few drops of xylene was added to the solvent trap covering the gap prior to each measurement. The linear viscoelastic region was found by conducting a strain sweep from 0.001 to 1% strain. The strain was set to 0.01% selected from the linear viscoelastic region. The angular frequency was set from 0.1 to 100 rad s^–1^ and the strain 0.01% was applied to measure the storage modulus (*G*′), as a measure of the solid-like, and the loss modulus (*G*″), as a measure of the liquid-like characteristics of viscoelastic gels. To ensure the reliability and reproducibility of the experiments, three independent samples of each *n*BA gel were prepared under the same condition and frequency sweep tests were performed on them using the same protocol.

## Results and Discussion

**Figure 2:**
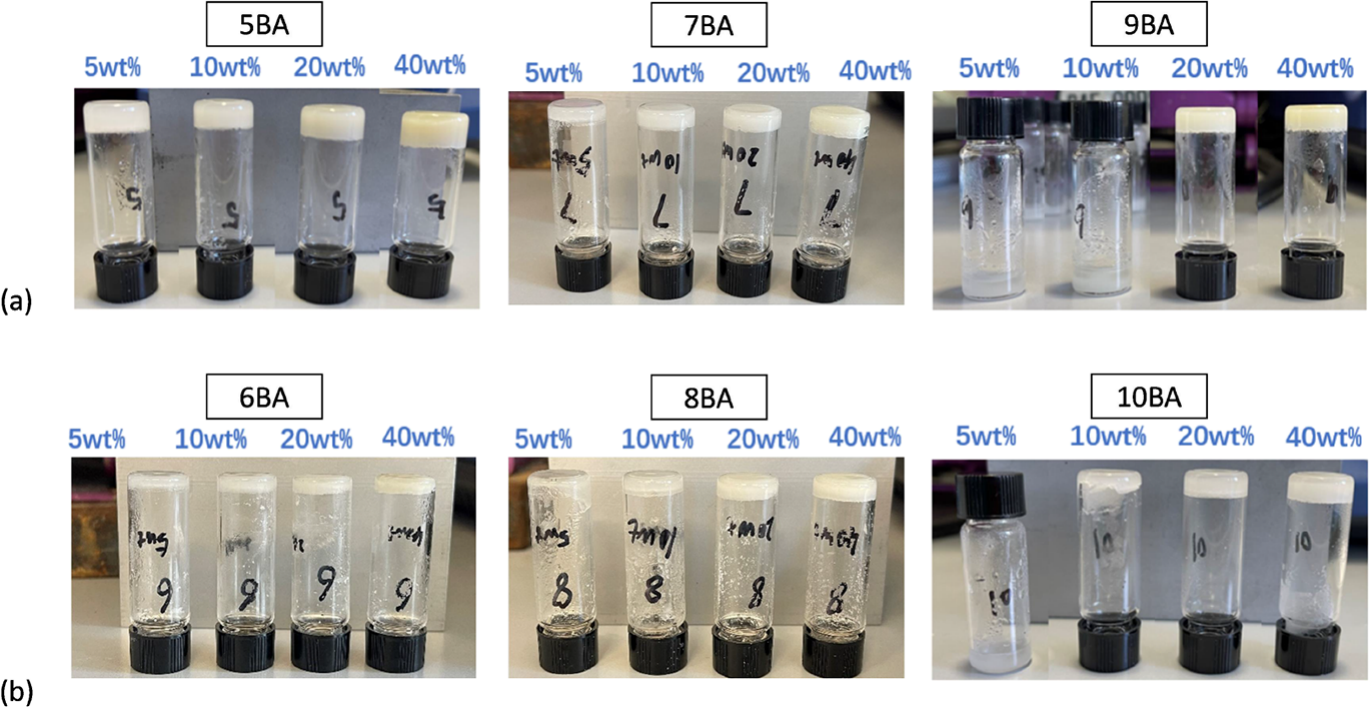
*n*BA gels at different concentrations (wt %): (a) odd *n*BA gels: 5BA gel, 7BA gel (all gels at different concentrations remain stable against the gravity), 9BA (gels at (5 wt %) and (10 wt %) start to flow as soon as the vial was inverted), (b) even *n*BA gels: 6BA gel (all concentrations remained stable against gravity), 8BA gel (at low concentrations (5 and 10 wt %) partially flowed), 10BA gel ((5 wt %) gel was too fluidic and could not bear the gravity upon inversion and (10 wt %) partially flowed).

The *n*BA gelators (5BA, 6BA,7BA, 8BA, 9BA, and 10BA) were dissolved in xylene at different concentrations (5, 10, 20, and 40 wt %) upon heating to obtain homogeneous and transparent solution without any clear supramolecular features remaining. After cooling to the room temperature, gels with more turbid appearance were produced. It was observed that the solubility of the *n*BA gelators is remarkably low at room temperature which is a common feature among similar low molecular weight gelators.^1^ The gelling capacity of the *n*BA gelators was qualitatively assessed by macroscopic observation of *n*BA gels upon tube inversion (Figure 2). In the odd series, 5BA and 7BA have formed stable gels at all given concentrations while 9BA can only form gel at 20 and 40 wt %. In the even series, 6BA gels were stable at all of the concentrations. Similar to the odd series, upon increasing the spacer length of gelators in the even group, the gelling capacity of the gelators decreases; 8BA and 10BA form gels at low concentrations (5 and 10 wt %) that are less stable against gravity. Screening the macroscopic appearance of the gels after 72 h did not show disintegration or change of color. Further characterizations on the gels, described below, were carried out within 24 h of gel preparation.

**Figure 3:**
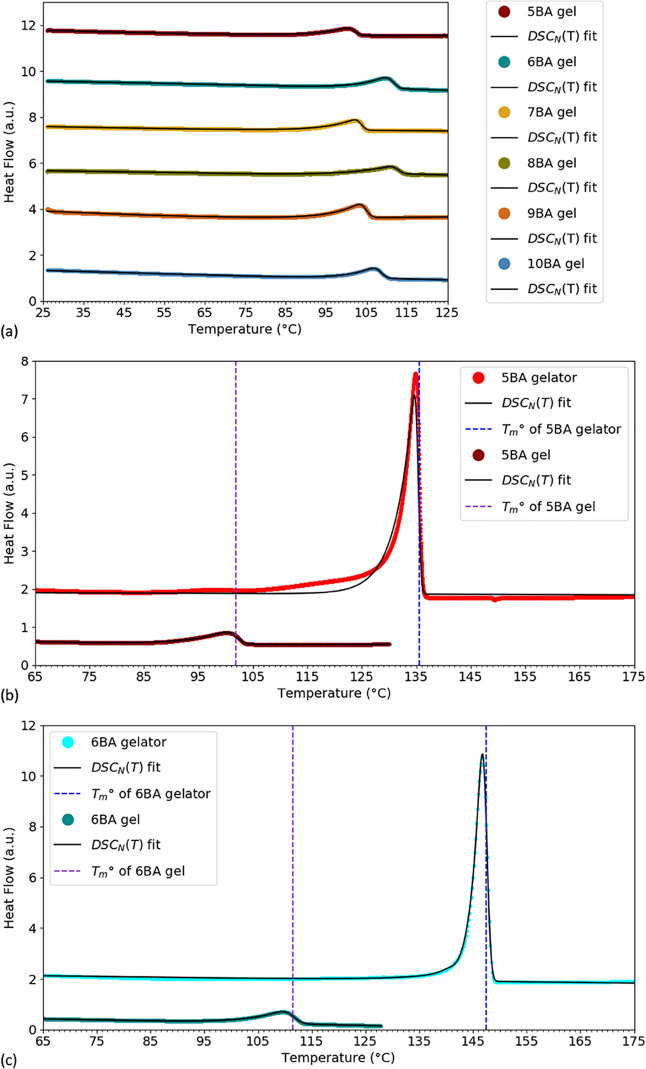
DSC thermogram of *n*BA gels (20 wt %) (curves were shifted vertically for clarity): (a) DSC_N_(T) function fitted to the second heating traces (endo up) measured at 5 K min^–1^ after calibration at the onset for the given sample weight and scan rate, (b) the second heating traces, fits, and *T*_m_^0^ of 5BA (as an example of the odd series) were compared in the gelator and gel states, (c) the second heating traces, fits, and *T*_m_^0^ of 6BA (as an example of the even series) were compared in the gelator and gel states, and the results in the gelator solid state are from our previous investigations.^41^

The *n*BA gels at 20 wt % were chosen for further analysis mainly because 20 wt % was the lowest concentration where all *n*BA gelators form stable gels as assessed by the vial inversion test. To identify the phase behavior of *n*BA gels 20 wt %, DSC measurements were conducted. Figure 3a shows a single endothermic transition in the second heating traces of the *n*BA gels which were heated from 25 to 130 °C (the first heating trace of 5BA gel (20 wt %) shows a double peak, as seen in Figure S1 in the Supporting Information (SI)). Compared to the thermogram of the *n*BA gelators in the solid state (Figure 3b,c), those endothermic transitions can be attributed to the melting of the *n*BA gels.^41^ Similar to the DSC traces of *n*BA gelators, the DSC_N_(T) function fits the second heating traces of all *n*BA gels (20 wt %) rather well (*R*^2^ > 0.95), yielding melting temperature (*T*_m_^0^) of the gels as listed in Table 1 (Figure S2 shows the fit features in more details and all the fit parameters of all *n*BA gels are available in Table S1, in the SI file).

**Table 1:** Melting Temperature (*T*_m_^0^) and Statistical Coefficient (*R*^2^) Obtained from the DSC_N_(T) Model Fit on the Experimental Curves of *n*BA Gels (20 wt %) Heated at 5 K min^–1^ after Calibration at the Onset for the Given Weight and Rate^a^

*n*BA gels (20 wt %)	*T*_m_^0^ (°C)	*R*^2^
5BA	101.92 ± 0.02	0.97
6BA	111.49 ± 0.03	0.96
7BA	103.60 ± 0.01	0.98
8BA	112.71 ± 0.01	0.96
9BA	104.82 ± 0.01	0.98
10BA	108.42 ± 0.04	0.95

a The error margins are the errors from the non-linear fitting.

As Figure 3b,c shows, the melting temperatures of 5BA and 6BA, as examples from odd and even series, respectively, are somewhat lower than the melting temperatures of their respective gelators in the solid state (approximately 35 °C lower). To gain more insight into the dissolution behavior of these gelators, we determined the solubility curves of these gelators. The solubility curve can be derived from DSC experiments via the following two methods:

1. The first approach is to plot the curve based on the *T*_m_^0^ obtained from the DSC_N_(T) model fit to the second heating traces of the gels at different concentrations which were measured by DSC (Figure S3).
2. The second approach is obtaining the curve from the cumulative integration of the melting transition in the second heating DSC trace of the gels (20 wt %). This method is described in more details in the Supporting Information (Figure S4).

**Figure 4:**
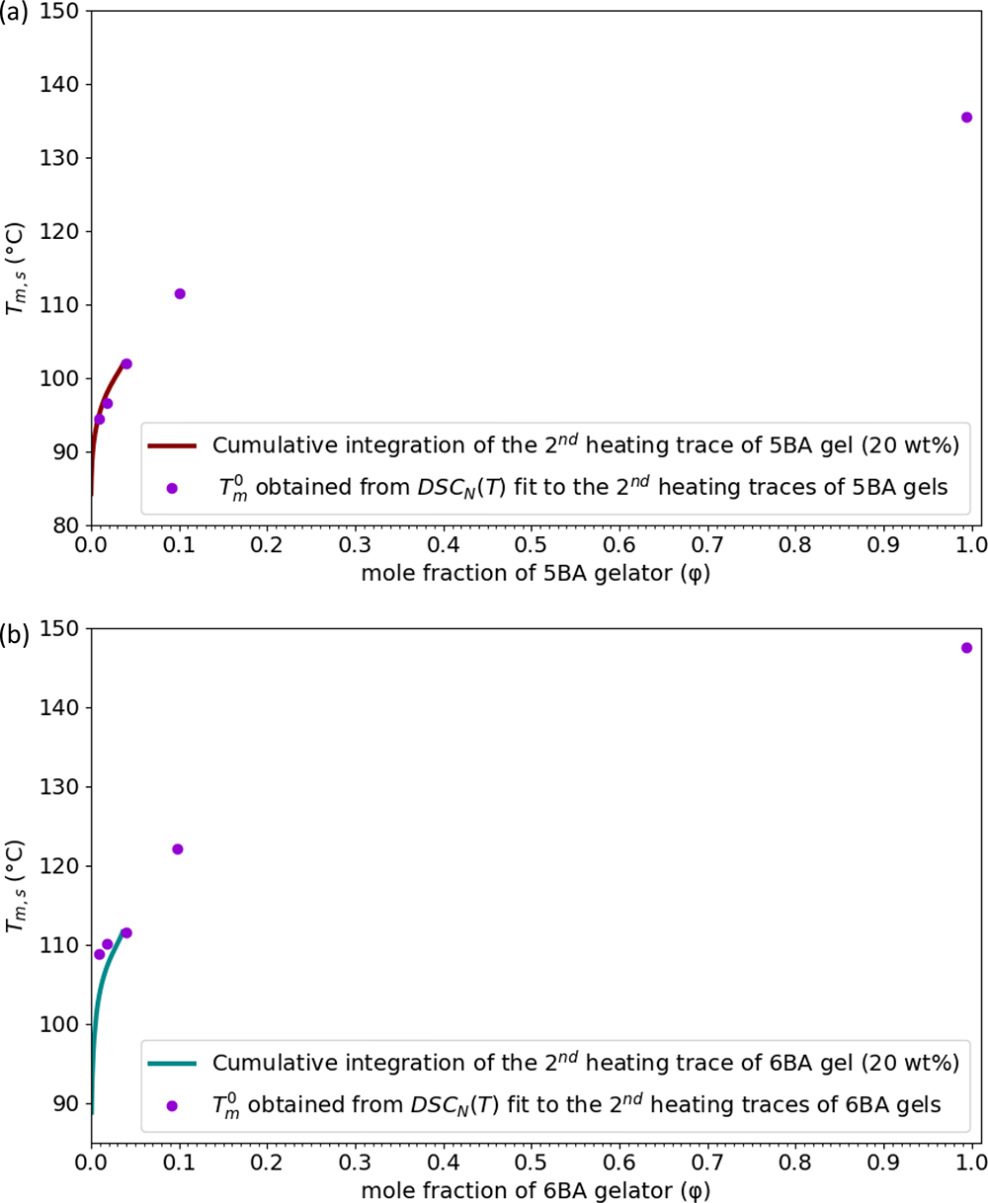
Solubility curves obtained from two different methods for (a) 5BA, (b) 6BA gelators showing the melting temperature of the systems versus the mole fraction of the gelators present in xylene as the solvent (in the case of both gelators, the mole fraction (φ = 1) is the pure gelator). The unit of temperature in the FHM model is (K); however, the curves were plotted based on the (°C) scale to be consistent with DSC thermograms, as shown in Figure 3.

Figure 4 shows the solubility curves for 5BA and 6BA gelators obtained from two different methods. The *T*_m_^0^ of 5BA and 6BA gels at different concentrations fit to the solubility curve obtained from the cumulative integration of their gels (20 wt %) up to φ = 0.040 and φ = 0.039, which are, respectively, the mole fraction of 5BA and 6BA in their 20 wt % gels (Figure 4).

To gain insight into the mechanism of dissolution of *n*BA systems, we combined the well-known Flory–Huggins theory for mixing with the thermodynamics of melting, briefly the FHM model, as described by eq 2 (the route we took to develop the FHM model is described in the Supporting Information).
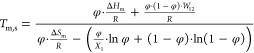
2

The FHM model combines the theories of melting, describing the thermodynamics of the phase behavior in the solid crystalline or melt state, and Flory–Huggins, which originally elaborates the thermodynamics of polymer solutions, explains the thermodynamics of *n*BA gelators melting in the presence of a solvent. The FHM model is based on thermodynamic parameters which describe how the melting point of a *n*BA gel (*T*_m,s_) changes with the volume fraction of the gelator (φ) (accordingly, (1 – φ) is the volume fraction of the solvent). The change in enthalpy and entropy of melting for a solid gelator is notated as Δ*H*_m_ and Δ*S*_m_ respectively. These two values are known from our previous research on the *n*BA gelators:^41^ the thermal properties of *n*BA gelators in the solid state, Δ*H*_m_ and *T*_m_^0^, were obtained via fitting DSC_N_(T) to the second heating DSC traces where the melting transition occurs. Given Δ*H*_m_ and *T*_m_^0^ of the gelators, Δ*S*_m_ is calculated from Δ*S*_m_ = Δ*H*_m_/*T*_m_^0^ for each *n*BA gelator (since Δ*G* is zero at the equilibrium). Therefore, the only adjustable parameters in eq 2 are *W*_12_ and *X*_1_: the enthalpy of dissolution (*W*_12_) is used to describe the interactions between the solvent and *n*BA gelator molecules. The parameter *X*_1_ is the degree of association of the molecules after melting.

**Figure 5:**
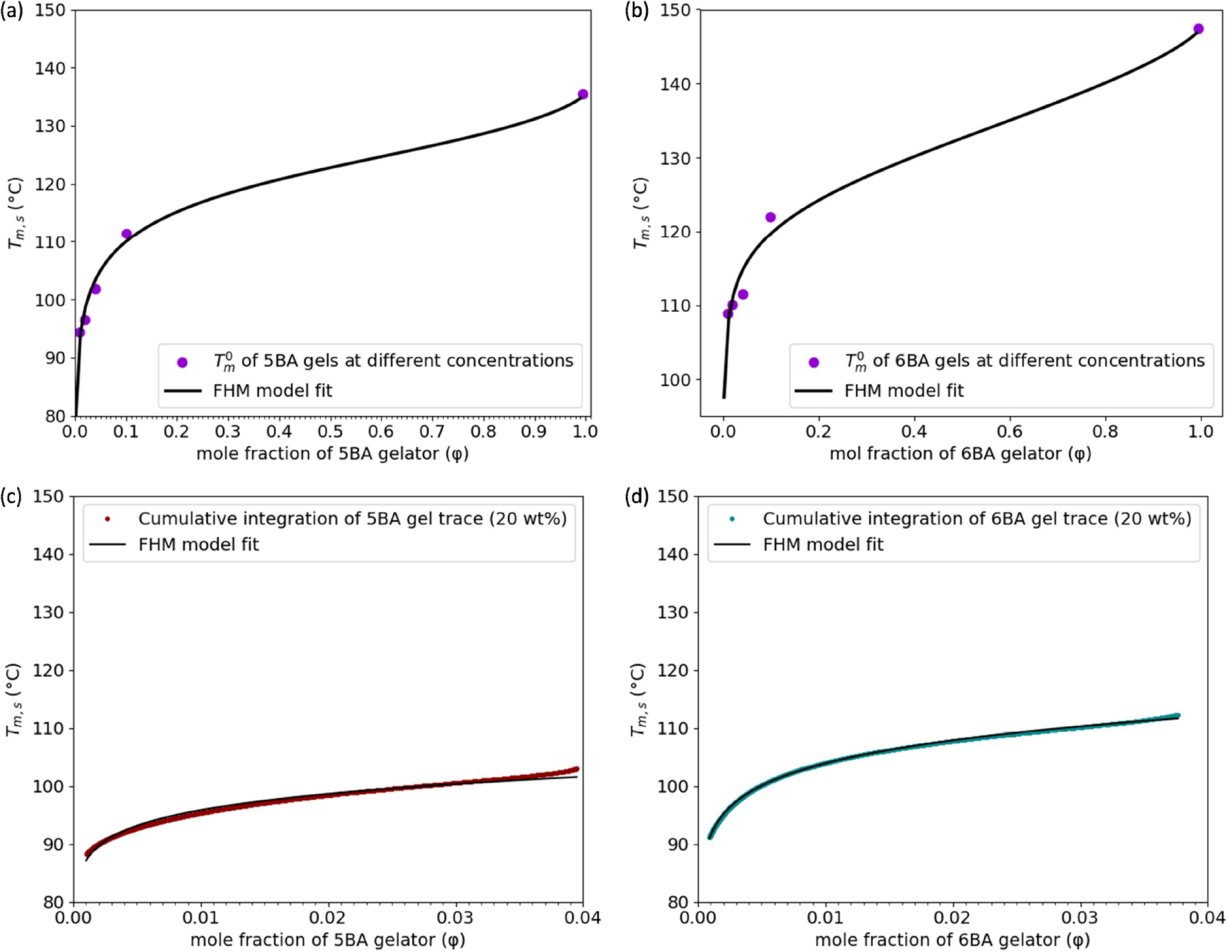
FHM model fitted to the solubility curves obtained from (a, b) *T*_m_^0^ obtained from the DSC_N_(T) model fit to the second heating traces of the gels at different concentrations for 5BA and 6BA, respectively; (c, d) from the cumulative integration of the second heating DSC traces of the 5BA and 6BA gels (20 wt %), respectively (the melting-dissolution curves for the rest of the gels can be found in the Supporting Information (Figure S6)). The unit of temperature in FHM model is (K); however, the curves were plotted based on the (°C) scale to be consistent with the DSC thermogram shown in Figure 3.

The FHM model fits to the solubility curves of 5BA and 6BA gels obtained from the first method quite well (Figure 5a,b). However, the second method provides the solubility curve with more data points. Figure 5c,d shows that the FHM model fits the melting–dissolution curve obtained from the latter method very well (*R*^2^ > 0.99 according to Table 2). At room temperature, the gelators dissolve negligibly in xylene; after raising the temperature close to their melting points (*T*_m_^0^), 101.92 ± 0.02 and 111.49 ± 0.03 °C, for 5BA and 6BA gels (20 wt %), respectively, more fractions of gelators start to melt, and above *T*_m_^0^, all the melted gelators start mixing with the solvent.

**Table 2:** Fitted Parameters and Statistical Coefficient of the FHM Model Fitted to the Cumulative Integration Curves of Second Heating DSC Traces of *n*BA Gels (20 wt %)^a^

*n*BA gels (20 wt %)	*W*_12_ (J mol^–1^)	*X*_1_	*R*^2^
5BA	–870.50 ± 9.28	1.96 ± 0.00	0.99
6BA	–939.10 ± 29.31	3.72 ± 0.03	0.99
7BA	–620.33 ± 9.04	2.69 ± 0.04	0.99
8BA	–155.33 ± 33.65	2.09 ± 0.01	0.96
9BA	–814.19 ± 17.29	3.13 ± 0.01	0.98
10BA	–1512.64 ± 33.51	2.11 ± 0.01	0.99

a The error margins are errors from the non-linear fitting.

*W*_12_ is negative and less than 2% of Δ*H*_m_ for all *n*BA gels (Table 2). It indicates that after melting of the gelators, there is a small affinity between *n*BA gelators and xylene to interact. In fact, the *W*_12_ term allows the fine-tuning of the initial slope of the solubility curves. *X*_1_ is larger than 1 for all *n*BA gelators, which indicates that after melting, there is still some degree of association between these molecules.

**Figure 6:**
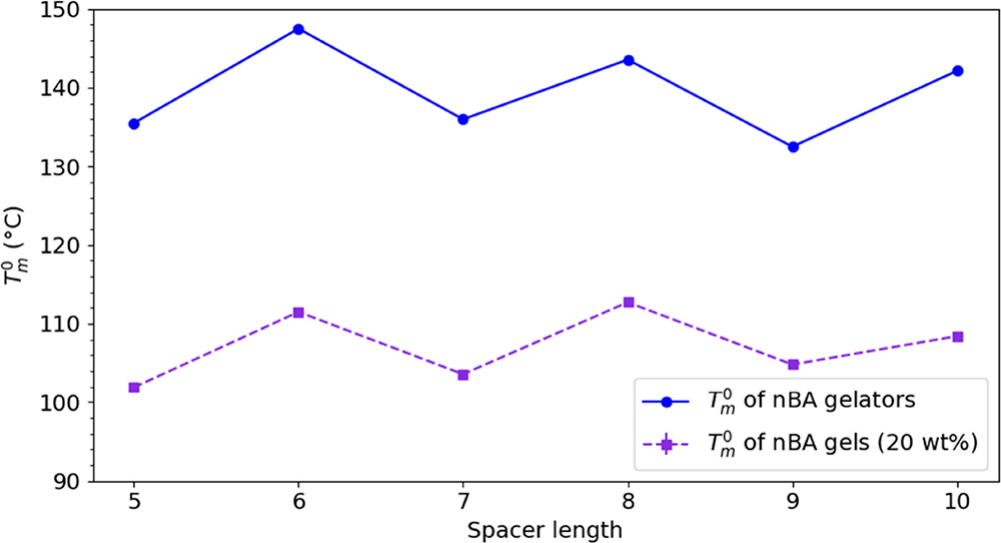
Melting temperature (*T*_m_^0^) of *n*BA gelators^41^ and the melting-dissolution temperature (*T*_m_^0^) of *n*BA gels (20 wt %) obtained from DSC_N_(T) fit to their second heating DSC traces.

The FHM model basically says that *n*BA gelators with a rather large Δ*H*_m_ are not inclined to dissolve because there is a Δ*H* penalty; thus, below the melting point, only a small concentration of the *n*BA gelator is dissolved in the xylene solvent. Once the gelator solid crystal melts, the gelator molecules mix with the solvent, which increases the entropy via the entropy of mixing. Therefore, the melting point of the system is reduced. As Figure 6 shows, the melting points of the pure *n*BA gelators are generally about 35 °C higher than their melting-dissolution temperatures in the presence of xylene, which is in agreement with FHM model theory. Other than that, the transition temperatures show a remarkably similar odd–even effect.

Like the solid *n*BA gelators, the *n*BA gels also show a clear odd–even effect in *T*_m_^0^ upon increasing the spacer length from 5 to 10 (Figure 5). *T*_m_^0^ of even *n*BA gels is generally higher than the odd ones, like *n*BA gelators, which is attributed to their molecular arrangement.^41^ In fact, odd *n*BA gelator molecules are in less favorable conformations, i.e., not at their lowest conformational energy to allow the H-bonding network to develop.^38^ Therefore, the odd members are under more internal stress. The even *n*BA gelators have higher melting points because the H-bonding is more regular as is achieved while keeping conformations close to the optimum lowest energy state. The question now arises whether the underlying crystal structures of the gels are actually similar to those of the pure gelator crystals? Figure 5 already indicates that this might be the case.

**Figure 7:**
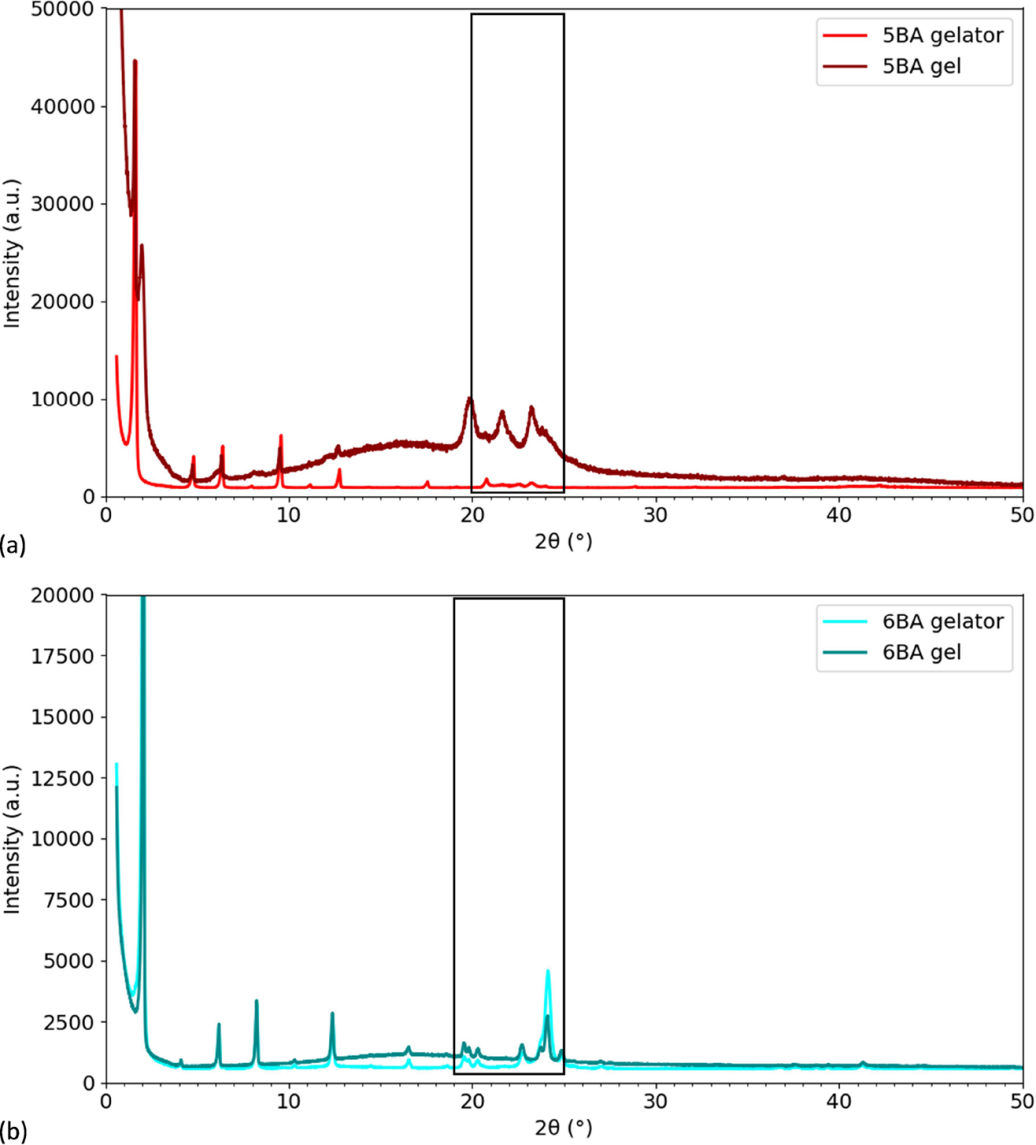
Observed XRD patterns of *n*BA gelators in the solid state and *n*BA gels (20 wt %): (a) 5BA, (b) 6BA, curves were normalized to the highest intensity and shifted vertically for clarity and solid boxes were added to guide eyes.^41^

To investigate the supramolecular arrangement of the *n*BA molecules in the gel state, XRD patterns of gels (20 wt %) were measured at ambient temperatures under the same conditions as the *n*BA gelators in the solid state (Figure 6). In our previous study, the XRD patterns of 5BA and 6BA gelators in the solid state were fully indexed, which revealed the pseudo-orthorhombic lattice for odd and a triclinic one for even *n*BA gelators.^41^ Unfortunately, the XRD patterns of gel samples could not be indexed independently due to a relatively low resolution of the reflections, and therefore, they were analyzed more globally by comparing them with the XRD patterns of the gelators in the solid state (Figures 7 and S7).

The general diffraction pattern of 5BA gel (20 wt %) is somewhat analogous to the 5BA gelator, the characteristic lamellar 00*l* reflections, except for an extra reflection at 1.99°(2θ) which is due to polymorphism.^1^ More notably though, the pattern of the 5BA gel (20 wt %) shows a much higher background intensity, and all reflections are strongly attenuated in intensity, indicating substantially less crystallinity in the 5BA gel. Moreover, the reflections in the 20–25°(2θ) region of 5BA in the gelator and gel state are quite different, which suggests that the side-by-side packing of the of 5BA molecules in the gel state is actually different to that in the solid state. The observed XRD patterns of the three odd *n*BA gels are shown in Figure S7d. The characteristic lamellar 00*l* reflections observed for the 5BA gel are observed for 7BA and 9BA gels as well, indicating a highly defined layer spacing. The shift to lower angles with increasing spacer length from 5BA to 9BA is due to an increasing *c*-axis length. The reflections in the 20–25°(2θ) range are different among odd gels, implying a slightly different lateral packing for odd *n*BA gels.

The diffraction patterns of the 6BA gel (20 wt %) show many similarities with the pattern of the pure 6BA compound in the solid state. The larger background and lower intensity of the reflections observed for 5BA gel (20 wt %) are also observed for the 6BA gel (20 wt %) compared to the 6BA pure gelator, but to a much lesser extent. The reflections of 6BA gel and solid gelators in the 1–19°(2θ) low angle and 19–25°(2θ) wide angle range are identical. This similarity suggests that the supramolecular arrangement of 6BA molecules in a 20 wt % gel is very similar to the molecular arrangement in the pure compound. The XRD patterns of the even *n*BA gels in Figure S7b show 00*l* reflections for all even *n*BA gels. The *c*-axis length increases with increasing the spacer length which leads to a low-angle shift of the 00*l* peaks. The reflections in the 19–25° (2θ) range are very similar for the even gels, implying that the lateral packing of 8BA and 10BA is very similar to that of 6BA.

**Figure 8:**
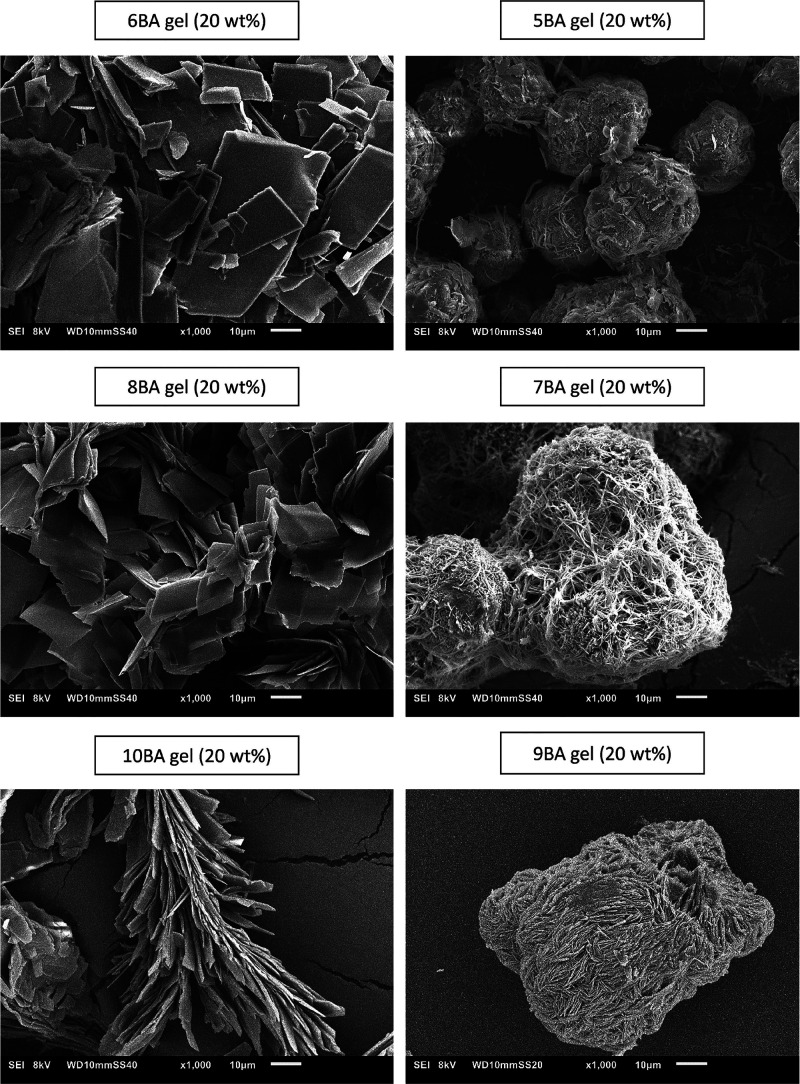
SEM images of *n*BA gels (20 wt %) depicting the sheetlike structure of even gels versus woven fibrous morphology of odd gels.

The similarity in XRD patterns of *n*BA gels and their gelators facilitates their analysis using the indexing of *n*BA gelators in the solid state;^41^ like the XRD pattern of *n*BA gelators, the 00*l* reflections are present in both odd and even *n*BA gels which indicates regular layer spacing even though some higher order reflections have relatively lower intensities. The gel state patterns also show a clear odd–even difference, suggesting the less favorable molecular conformation of odd molecules in the gel state. SEM was used to investigate the effect of the supramolecular structure on the gel morphology. As SEM images of *n*BA gels(20 wt %) in Figure 8 show, even *n*BA gels exhibit sheetlike crystals which are clearly distinguishable from the woven fibrous-like structure of odd *n*BA gels. This can be attributed to the packing model proposed for the even *n*BA molecules in the solid state;^41^ the stacking of several layers of molecules with the tilted lamellar geometry can self-assemble as sheetlike microcrystals observed for all even *n*BA gels. Odd *n*BA gels show three rather distinct morphologies which might be in line with their different XRD reflections in the 20–25°(2θ) region (Figure S7d); the 5BA gel shows a combination of woven fibers and spherical structures which is in agreement with the first two 00*l* reflections in its XRD pattern while 7BA and 9BA show a more uniform woven fiber structure. This variation in the microstructures of odd *n*BA gels is in reasonable agreement with the different microstructures, as displayed by their XRD patterns. A question might be whether these microstructural differences between odd and even *n*BA gels are reflected in their rheological properties.

**Figure 9:**
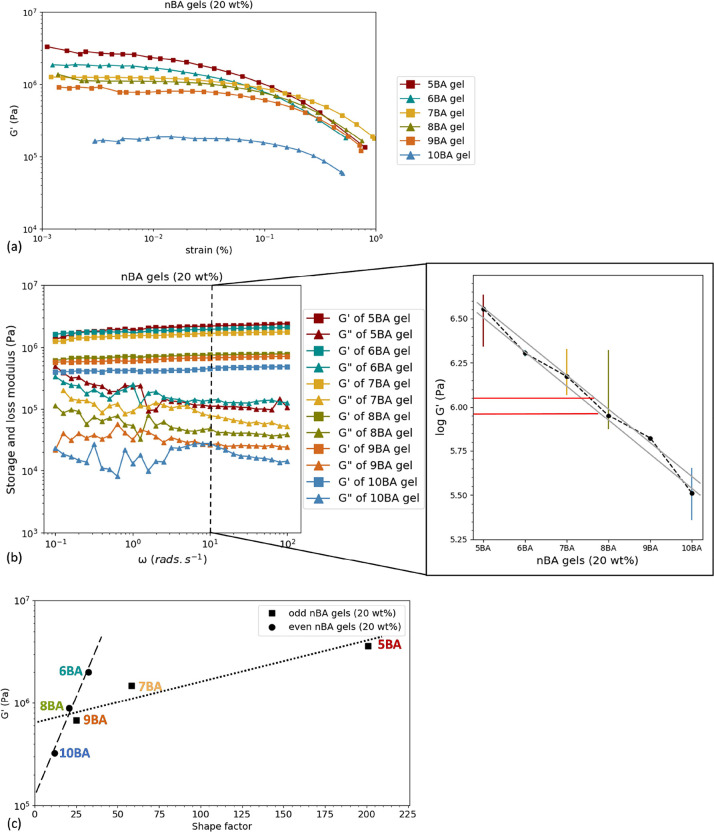
Rheological measurements on *n*BA gels (20 wt %): (a) amplitude-sweep measurements from 0.001 to 1% strain rate at a constant frequency of 1 Hz, (b) evolution of *G*′ and *G*″ as a function of angular frequency (ω): the subset shows the change in storage modulus (*G*′) with the spacer length in *n*BA gels (20 wt %), the red solid lines were added to guide eyes for odd–even alternation amounting to about 25% deviation from the overall (between the gray guidelines), (c) change of *G*′ with the shape factor of woven fibers and sheets in odd and even *n*BA gels (20 wt %), respectively.

To study the flow and deformation properties of *n*BA gels, initially, oscillatory rheological measurements (amplitude-sweep tests) were conducted on *n*BA gels (20 wt %). For all these gels, the elastic component (*G*′) dominates over the viscous component (*G*″) at small applied shear and reaches a plateau in the linear response viscoelastic region (LVR). As shown in Figure 9a, the yield strain is independent of the spacer length and for all *n*BA gels, it is below 1%, resulting in a much smaller LVR compared to hydrogels in general.^42^ The *n*BA gels are remarkably strain-sensitive compared to many cross-linked gels and other supramolecular and complex fluid systems that can often withstand strains 10–100% strain if not more. This observation in itself may indicate that bisamide organogelator gels might be highly sensitive to strain and strain history, which makes reliable rheological characterization quite challenging. Note that even at strains around 10^–3^% there is still some, admittedly mild, amplitude dependence, a strain of 1 part in 100,000 would normally be considered totally safe within the linear region.

Once LVR for every *n*BA gel was established, at the low strain of 0.01% in the LVR, frequency sweep experiments were conducted to evaluate the storage and loss moduli of the *n*BA gels (Figure 9b). Under this condition, the storage modulus of *n*BA gels increases only very slightly with frequency, so the gel does not have any appreciable internal dynamics. Clearly, at the probed timescales, the gel network fails to rearrange and shows an elastic behavior. At all frequencies, *G*′ > *G*″, which indicates the gel behavior for all *n*BA gels (20 wt %). Interestingly, *G*″ decreases slightly with increasing ω while one could anticipate an increasing trend based on *G*″ = η·ω.

The relation between storage modulus (*G*′) and angular frequency can be explained by the simplified power law equation below:^43^
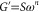
3

In eq 3, *S* is a material-specific constant, ω is the angular frequency, and *n* is the viscoelastic exponent. In our test, *n* is close to 0 for all *n*BA gels, which confirms their solid-like behavior where *G*′ is invariant of the measured frequency while depending on the material-specific constant (*S*); the higher the *S* value, the higher the gel network modulus.

To compare the modulus of *n*BA gels, the values of *G*′ at a constant frequency (ω = 10 rad·s^–1^) for all gels were selected (Figure 9b). The storage modulus (*G*′) of *n*BA gels (20 wt %) decreases by increasing the spacer length which is in a good agreement with the table-top rheology trend as observed from inverting the vials. Quantitative rheological measurements show that this decrease is not linearly related to the spacer length, but a slight odd-even alternation is observed, amounting to about 25% deviation from the overall trendline.

**Figure 10:**
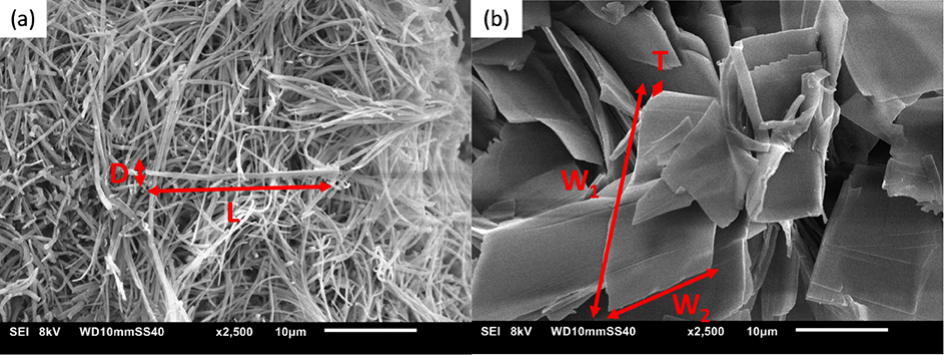
Schematic assignment of dimensions of (a) woven fibers in odd *n*BA gels (*L* ≫ *D*), (b) sheets in even *n*BA gels (*W*1 ≈ *W*2 and *W* ≫ *T*).

The relation between the gel morphology and rheological properties can be explained with inspiration by the Halpin–Tsai model which discusses the effect of the particle aspect ratio on the modulus of a composite material.^44^ The Halpin–Tsai model is based on the work by Hill, Kerner, and Hermans. It is often considered as a semi-empirical model. However, it has some unique features, such as taking the geometry of the filler in a composite into account that are absent in other models.^45^ Although applying this model to the *n*BA gel systems is not fully justified, we found the model valuable to investigate the effect of particle geometry on the rheological properties of the gels. It is reasonable to surmise that a *n*BA gel is a type of composite material which consists of two components, a space filling 3D gel network and the solvent matrix with some molecularly dissolved gelator at low concentrations. To justify the application of the Halpin-Tsai model, the matrix would be required to have sufficient stiffness for stress transfer, and the gel fibrils or sheets should act as a reinforcement, without taking any effect of fibril or sheet cross-linking into account. This evidently is not quite what we imagine an organogel to look like because the matrix might be a viscous fluid and the cross-links are the essence of what we might consider a required feature for a gel. The Halpin–Tsai model is given in eq 4:
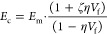
4
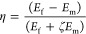
5where *E*_c_ refers to the storage modulus of the composite material, *E*_f_ refers to the modulus of the fiber, and *E*_m_ refers to the modulus of the matrix material. *V*_f_ is the fiber volume fraction and ζ is the reinforcing efficiency or the shape factor. The shape factor (ζ) is determined by the fiber geometry, molecular arrangement, packing fraction, and loading conditions.^46^ η is the stress-partitioning factor (eq 5). Assuming that the reinforcement only happens along the principal fiber direction, the formula of ζ_O_ for fibers with circular or rectangular cross-section is given as eq 6:
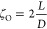
6where *L* is the length of the fiber in its elongated direction and *D* is the diameter of the circular or width of the rectangular cross-section in the rodlike fibers (Figure 10a). The shape factor for a sheet-like structure (ζ_E_) is given by eq 7, where *W* refers to the average width (*W*) of the sheets and *T* refers to the thickness of the sheets (Figure 10b):
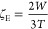
7

If we now substantially simplify the Halpin–Tsai in eq 4 for *E*_f_ ≫ *E*_m_ and low fill fraction, the stiffness is obtained from eq 8:
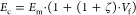
8

As a result, the storage modulus of the composite material is linearly proportional to the aspect ratio of the fillers at the same gel concentration based on eq 8. It is worth observing that this equation is actually similar to the Einstein law for viscosity (eq 9).
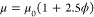
9where μ is the viscosity of the homogeneous suspension fluid, and the Einstein coefficient is 2.5 which is related to the spherical shape of the particles, μ_0_ is the liquid viscosity, and ϕ is the particle volume fraction.

Figure 9c shows that the storage modulus of even and odd *n*BA gels are indeed linearly proportional to the shape factor of sheets and fibers, respectively (ζ_E_ and ζ_O_)_,_ albeit with a rather different slope. Because the Halpin–Tsai model is only partially applicable to the gel system at hand, due to the physically cross-linked network, it should not be expected to give a very accurate prediction of the gel behavior. However, it does serve to underline the effect of the morphology on the gel properties which obviously deserves more extensive analysis.^47,48^

Another approach we considered was to estimate whether the organogels could be modeled as an open or closed cell foam, where the stiffness is wholly due to the “sponge” of the solid gelator, and the solvent/solution contribution would be negligible (eq 10). For a closed-cell foam, the modulus is linear with the volume fraction, whereas for an open-cell foam, the modulus is proportional to the square of the volume fraction, i.e., *p* = 1 and *p* = 2, respectively.^49,50^
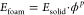
10

If we fill this in using *E*_solid_ is about 3 GPa and *f* = 0.2, we end up with gel moduli in the 120–600 MPa, a regime which is far too high. Some reduction to these values due to some Brownian motion of the gel fibrils and sheets might improve things slightly, but it is clear that quite more work needs to be done which is outside the scope of the current study.

## Conclusions

To study the gelation behavior of bisamide gels, *n*BA gelators with simple structures where *n* is the spacer length between amide groups (varying from 5 to 10) with 17 carbons at each side of the amide groups (C17) were used. The relatively simple chemical structure of these gelators makes them ideal as model compounds to study their supramolecular assembly, gelation, and gel properties of bisamide molecules in general. The spatial arrangement of gelator molecules in the gel state has been compared with the molecular arrangement in the solid state. The microscopic properties of the gels and their impacts on the final gel rheological properties have been investigated.

The instant table-top tube inversion test indicated that gelling capacity of *n*BA gelators decreases upon increasing the spacer length in both odd and even gels; as a result, around at least 20 wt % gelators is required for the formation of homogenous stable gels suitable for the further analyses. *T*_m_^0^ obtained from fitting of DSC_N_(T) to the DSC traces of *n*BA gels is about 35 °C lower than *T*_m_^0^ of the respective *n*BA gelators. This substantial difference is explained via the FHM model, combining the theories of Flory–Huggins and Gibbs free energy of melting. The FHM model fitting the melting-dissolution curves of *n*BA gels includes the entropy of the mixing term changing the level of order as the gelators transform from the solid state to the melted and dissolved state. Lower *T*_m_^0^ and Δ*H* of *n*BA gels compared to the *n*BA gelators are primarily caused by the entropy of mixing. The XRD patterns of the *n*BA gels (20 wt %) show 00*l* reflections matching with the respective pure gelators in the solid state, implying a regular structure. The similar reflections in 19–25°(2θ) region of even *n*BA gels and gelators were observed, which confirms the observation of SEM images: the sheetlike microstructures of even gels are in a reasonable agreement with the analogous lamellar spacing of the even gelators in the solid state. In contrast, the 20–25°(2θ) region of odd *n*BA gels was quite different to the gelator solid state and also in comparison to each other. The supramolecular arrangement of these molecules in the gel state apparently is distinctly different for the odd and even *n*BA gelators but the complete analysis of this is outside of the scope of this study. It is worth noting the very close trends in the melting temperatures suggest that the differences might still be rather despite substantial changes in the XRD patterns. Concerning the observed mixed morphologies of woven fibers and spheres found in the SEM images of odd *n*BA gels, this could indicate some polymorphism. The relation between the microstructure and rheological properties was explained using some inspiration from the Halpin–Tsai model, and indeed, the results were also compared to foam mechanics. Here, the *n*BA gels are considered as composite materials consisting of two components, the gel network crystals and the entrapped solvent with a certain degree of stiffness. The change of the storage modulus (*G*′) with the shape factor of woven fibers and sheets in *n*BA gels (20 wt %) indicates an odd–even effect. The *G*′ of even and odd *n*BA gels are linearly proportional to ζ_E_ and ζ_O_, respectively, which is in some agreement with the simplified Halpin–Tsai model; the difference in slope, however, seems unexplained as yet. Analogously, we found that foam mechanics substantially overestimates the gel moduli again, indicating that more subtle approaches to bisamide organogelator mechanics are required. Once such a rheological model is established, it will allow for more rapid optimization of gel properties and will provide a solid theoretical framework to build on.

## Supporting Information Available

More information about the methodologies and the analytical data for all *n*BA gels (PDF)
