# Supplementary material for: Supramolecular Arrangement and Rheological Properties of Bisamide Gels
Source: Langmuir. 2023 Jul 26;39(31):10913–24. doi: 10.1021/acs.langmuir.3c01100 (PMC10413945; doi:10.1021/acs.langmuir.3c01100)
Supplement: Supplementary file 1 — la3c01100_si_001.pdf [file la3c01100_si_001.pdf]

Supporting information

Supramolecular arrangement and rheological properties of bisamide gels

Authors: Elmira Ghanbari[1], Zian Chen[1], Pooja Padmanabhan[1], Stephen J. Picken[1], Jan H. van Esch[1,*]

[1]Advanced Soft Matter (ASM) group, Chemical engineering department, faculty of applied science (TNW), Delft University of Technology, 2629 HZ, Delft, The Netherlands
*Corresponding Author: Jan H. van Esch

Email: j.h.vanesch@tudelft.nl

Here, more information about the methodologies and the analytical data for all nBA gels are provided.

DSC thermogram of 5BA gel (20 wt%)

The DSC thermogram of 5BA gel (20 wt%) is shown in Figure S1. The first heating trace shows two peaks for the melting-dissolution transition while the second heating trace (Figure S2) shows a single endothermic peak. To obtain T_m^0, $DSC_N(T)$ was fitted to the normalized traces. The fit parameters are listed in Table S1.

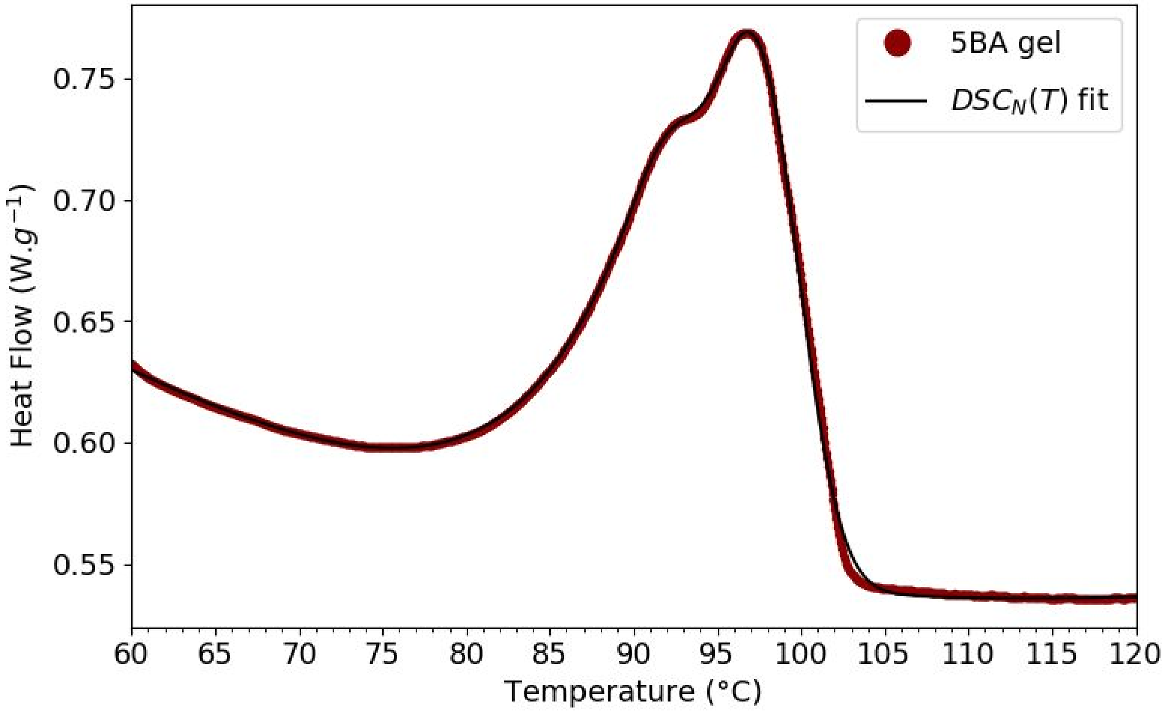

Figure S1. First heating DSC trace of 5BA gel (20 wt%) showing a double peak at the melting-dissolution transition.

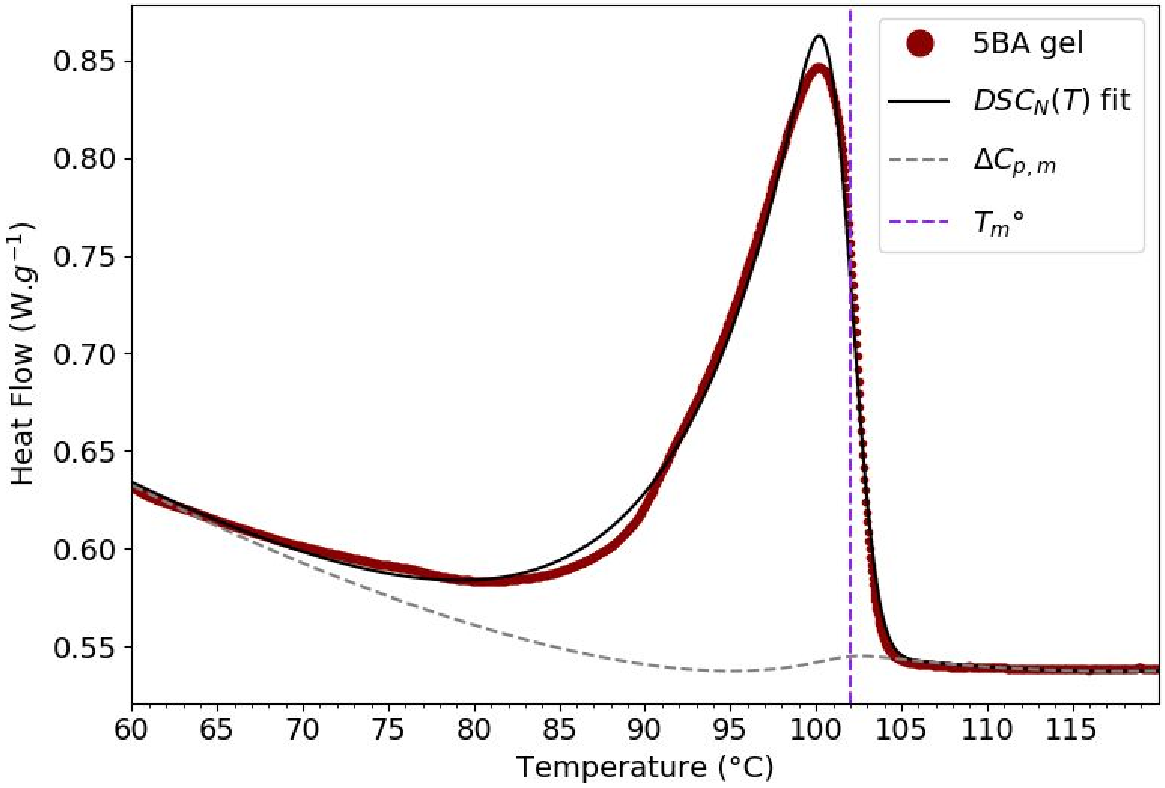

Figure S2. The second heating, just as an example of a nBA gel, to show how T_m^0 and $\Delta C_{p,m}$ are obtained from fitting of the $DSC_N(T)$ function to the experimental trace.

Table S1. Fitted parameters, standard deviations, and goodness of fit obtained from fitting $DSC_N(T)$ model to the 2nd heating DSC traces of nBA gels (20 wt%) heated at 5 $K.min^{-1}$ after calibration at the onset for the given weight and rate (the error margins exclusively are the errors from the non-linear fitting itself).

Parameter	5BA gel	6BA gel	7BA gel	8BA gel	9BA gel	10BA gel
ΔH ($J.g^{-1}$)	34.32±0.02	53.88±0.06	51.84±0.01	52.44±0.02	64.08±0.01	47.64±0.07
T_m^0 (°C)	101.92±0.02	111.49±0.03	103.60±0.01	112.71±0.01	104.82±0.01	108.42±0.04
α (K^{-1})	0.17±0.00	0.15±0.00	0.14±0.00	0.11±0.00	0.15±0.00	0.16±0.00
β (K^{-2})	0.31±0.01	0.31±0.02	0.31±0.01	0.35±0.01	0.44±0.01	0.32±0.03
$\Delta C_{p,m}$ ($W.g^{-1}.K^{-1}$)	0.01±0.00	0.03±0.01	0.03±0.00	0.13±0.00	0.04±0.00	0.05±0.01
B ($W.g^{-1}$)	0.55±0.00	0.20±0.01	0.39±0.00	0.39±0.00	1.59±0.00	0.44±0.01
C ($mW.g^{-1}.K^{-1}$)	-7.97±0.03	-5.50±0.02	-1.43±0.03	-3.43±0.07	0.09±0.04	-4.40±0.28
D ($mW.g^{-1}.K^{-2}$)	0.03±0.00	-0.02±0.00	0.01±0.00	0.01±0.00	0.05±0.00	0.00±0.00
R^2	0.97	0.96	0.98	0.96	0.98	0.95

FHM model development

The underlying principle of melting theory explains that the Gibbs free-energy (per volume) of the solid ($G_{s,v}$) and liquid state ($G_{l,v}$) at the equilibrium melting is the same, therefore $\Delta G=0$ at the melting point (Equation S1). Thus, the melting point is obtained from Equation S2 where T_m is the equilibrium melting point and ΔS and ΔH are the change in entropy and enthalpy respectively.

$$\Delta G = G_{l,v} - G_{s,v} = \Delta H_v - T\Delta S_v \quad \text{Equation S1}$$

$$T_m = \frac{\Delta H_v}{\Delta S_v} \quad \text{Equation S2}$$

Flory-Huggins theory is frequently used for polymer solutions (Equation S3) where φ_1 and φ_2 are volume fractions of polymer (gelator in the case of LMWG gels) and solvent ($\varphi_1 = \frac{N_1}{N_1 + xN_2}$, $\varphi_2 = \frac{xN_2}{N_1 + xN_2}$), N_1 and N_2 are the number of moles of polymer/gelator and solvent components respectively. The χ parameter is a free energy parameter thus including entropic component. Accordingly, W_{12} describes the enthalpy interaction between the solute and solvent ($W_{12} = \chi RT$). The parameter X_1 is the degree of polymerization or degree of association of the gelator molecules in solution (in the vicinity of the gel melting-dissolution transition).

$$\Delta G_m = RT(n_1 \ln\varphi_1 + n_2 \ln\varphi_2 + n_1\varphi_2\chi) \quad \text{Equation S3}$$

FHM model combines the free energy of melting with the free energy of dissolution into the same framework in Equation S4.

$$\Delta G = \varphi(\Delta H_m - T\Delta S_m) + RT((\frac{\varphi}{X_1})\ln\varphi + (1-\varphi)\ln(1-\varphi) + \varphi(1-\varphi)W_{12}) \quad \text{Equation S4}$$

Upon dissolution, ΔG becomes 0 which ultimately yields in equation S5, where the $\frac{\varphi(1-\varphi)w12}{R(\varphi\ln(\varphi)+(1-\varphi)\ln(1-\varphi))}$ term is derived from Flory-Huggins, and the $\frac{\varphi\Delta Hm}{\varphi\Delta Sm}$ term is derived from the melting theory of the pure compounds.

$$T_{m,s} = \frac{\varphi\frac{\Delta H_m}{R} + \frac{\varphi(1-\varphi)W_{12}}{R}}{\varphi\frac{\Delta S_m}{R} - (\frac{\varphi}{X_1}\ln\varphi + (1-\varphi)\ln(1-\varphi))}$$ Equation S5

Solubility curves of nBA gels

1. First method based on T_m^0

The solubility curves for 5BA and 6BA gels, as representatives of odd and even gels, were plotted using Tm0 of gels at different concentrations which were obtained from fitting DSCN(T) model to the second heating trace of 5BA and 6BA gels at different concentrations (Figure S3). It is worth to note that the DSC traces of 5BA gels at different concentrations show that the increase of both the melting temperature and enthalpy is proportional to the increase in the concentration of 5BA gels (Figure S3a). However, this is not the case for 6BA gels (Figure S3b). This could have been caused due to not scooping out homogeneous samples for DSC measurements, although gels looked uniform, or due to the solvent evaporation during sample preparation, although hermetically sealed pans were used for the measurements.

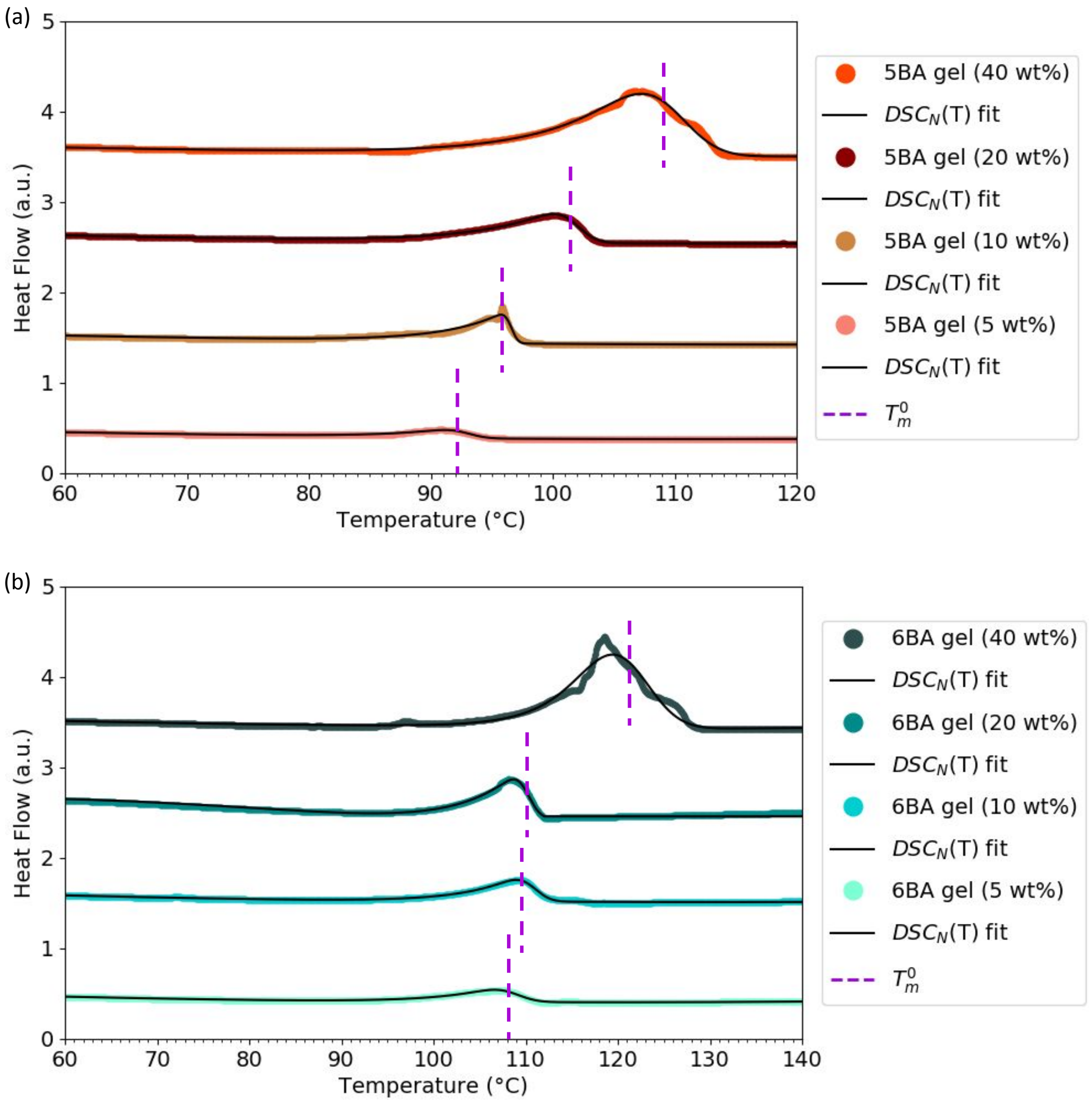

Figure S3. DSC_N(T) model fitted to the second heating traces of: a) 5BA gels, b) 6BA gels at different concentrations yielded T_m^0 of the gels (curves were shifted vertically for clarity).

2. Second method based on the cumulative integration of DSC trace

The second approach that we implemented was to obtain the solubility curve based on the cumulative integration of the melting transition of the nBA gel (20 wt%). The underlying rationale of this method is that DSC technique measures the heat uptake for an endothermic transition while the temperature increases. The consumed heat in DSC corresponds to the necessary heat for dissolution of a fraction of the gel crystals. Evidently the total heat taken up in the DSC heating trace corresponds to the heat consumed during the dissolution process.

As Figure S3 shows, the nearly similar shape of the DSC curves for nBA gels at different concentrations allows to obtain the solubility curve of the gel from a single DSC trace. In this method, the cumulative integration of the peak area in the single DSC trace (20 wt%) up to the endpoint, the maximum concentration, is calculated. At low temperatures of the heating trace, the solubility of the gelator is assumed to be reasonably close to zero and at the end of the transition peak all gelator molecules are dissolved. By assuming that the uptake of the heat is linearly proportional to the concentration, as heat capacity hardly changes over a small temperature window (on average 20 °C) [13NS], the solubility curve of 5BA as an example is obtained using the integration of the DSC trace (Figure S4a) and inversion of x and y axis (Figure S4b).The resulting solubility curve for 5BA is plotted in Figure S4c.

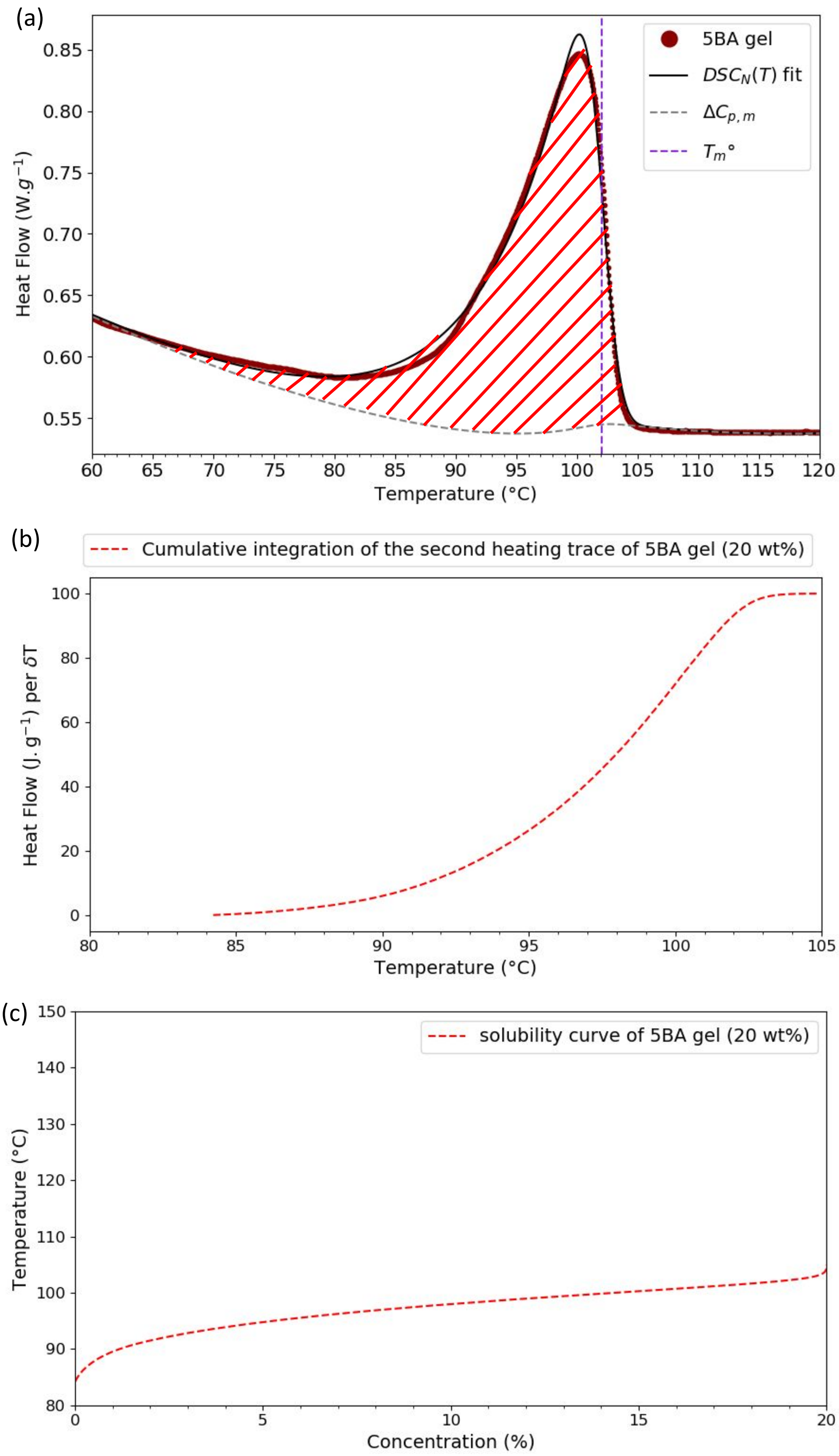

Figure S4. Cumulative integration method used to plot the solubility curve for 5BA gel as example: a) the second heating DSC trace of 5BA gel (20 wt%) and the integration of the peak region, b) cumulative integration of 5BA DSC trace versus temperature, c) solubility curve showing temperature change with change in concentration.

Microstructure evolution of gels with change in the concentration

Increasing the concentration of 5BA gelators in xylene results in a microstructural evolution in the gel state; the woven structure at 5 wt% turns into the mixture of woven and spheres at 10 wt% and 20 wt%. The woven fibers change to sheet-like structure at 40 wt% (Figure S5). For 6BA gels at all concentrations, sheet-like structures are observed which are more densely aggregated at lower concentrations. At higher concentrations, larger sheets have formed.

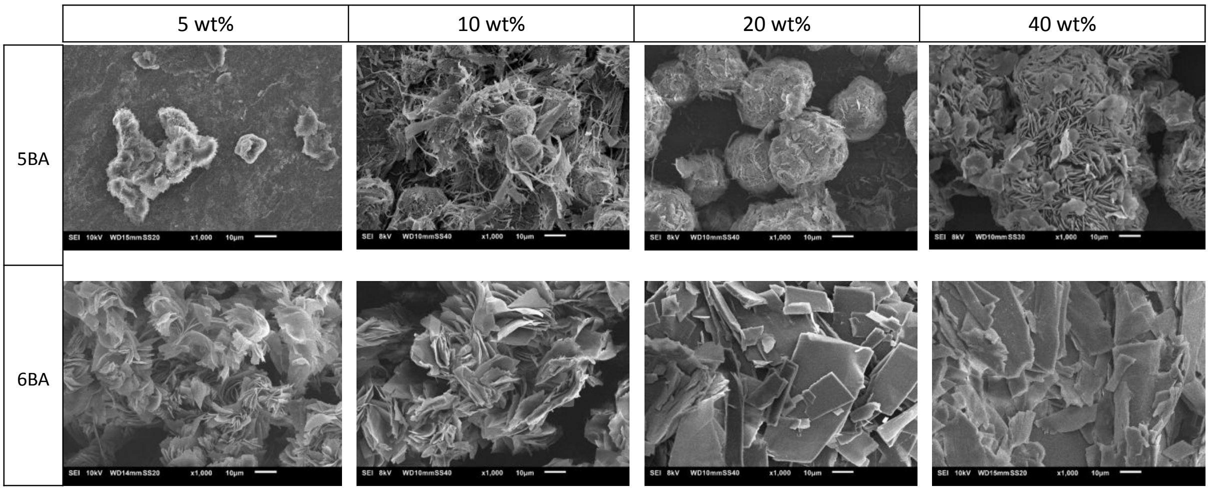

Figure S5. SEM images of 5BA and 6BA gels at different concentrations showing the microstructural evolution upon increasing the concentration from 5 wt% to 40 wt%.

FHM model fitted to the solubility curves of nBA gels (20 wt%)

Figure S6 shows that FHM model fits to the solubility curves obtained from the cumulative integration of the 2nd heating DSC traces of nBA gels (20 wt%).

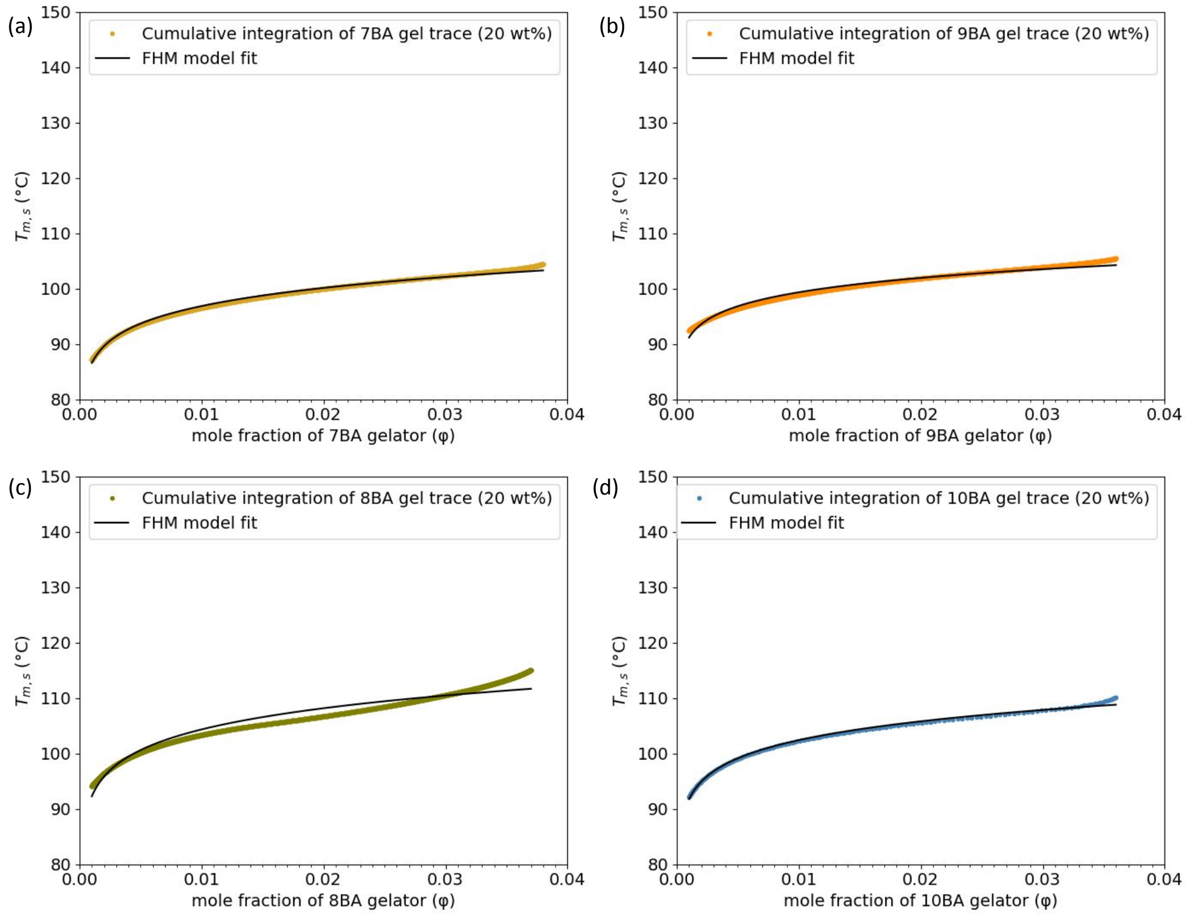

Figure S6. FHM model fitted to the solubility curves of a) 7BA, b) 8BA, c) 9BA, and d) 10BA gels (20 wt%). These solubility curves were obtained from the cumulative integration of DSC traces from the onset up to 100 % dissolution of the gels.

XRD patterns of nBA gels (20 wt%) in comparison with their gelators in the solid state

Using the indexing of the nBA gelators in the solid state from our previous research [37], the *00l* reflections are present in both odd and even nBA gels (Figure S7) which indicate the regular layer spacing even though some higher order reflections have relatively lower intensities [37]; the d-spacings of the peaks corresponding to *(00l)* family of planes representing the lamellar structure of the molecules which is known as the one-dimensional array of molecules stacked on top of one another with a constant bilayer thickness [1], [2]. The *c*-axis length increases with increasing spacer length which leads to a low-angle shift of the *00l* peaks. This similarity between the packing in the gel phase and the solid phase is seen in many bisamide and biscarbamate systems; the monocarbamates studied by Sundararajan et al. observed structural similarities between the gel phase and solid state of the gelators [3]. Terech et al. reported similar X-Ray diffraction patterns in organogels, xerogels and crystalline powder of 12-hydroxystearic acid [4].

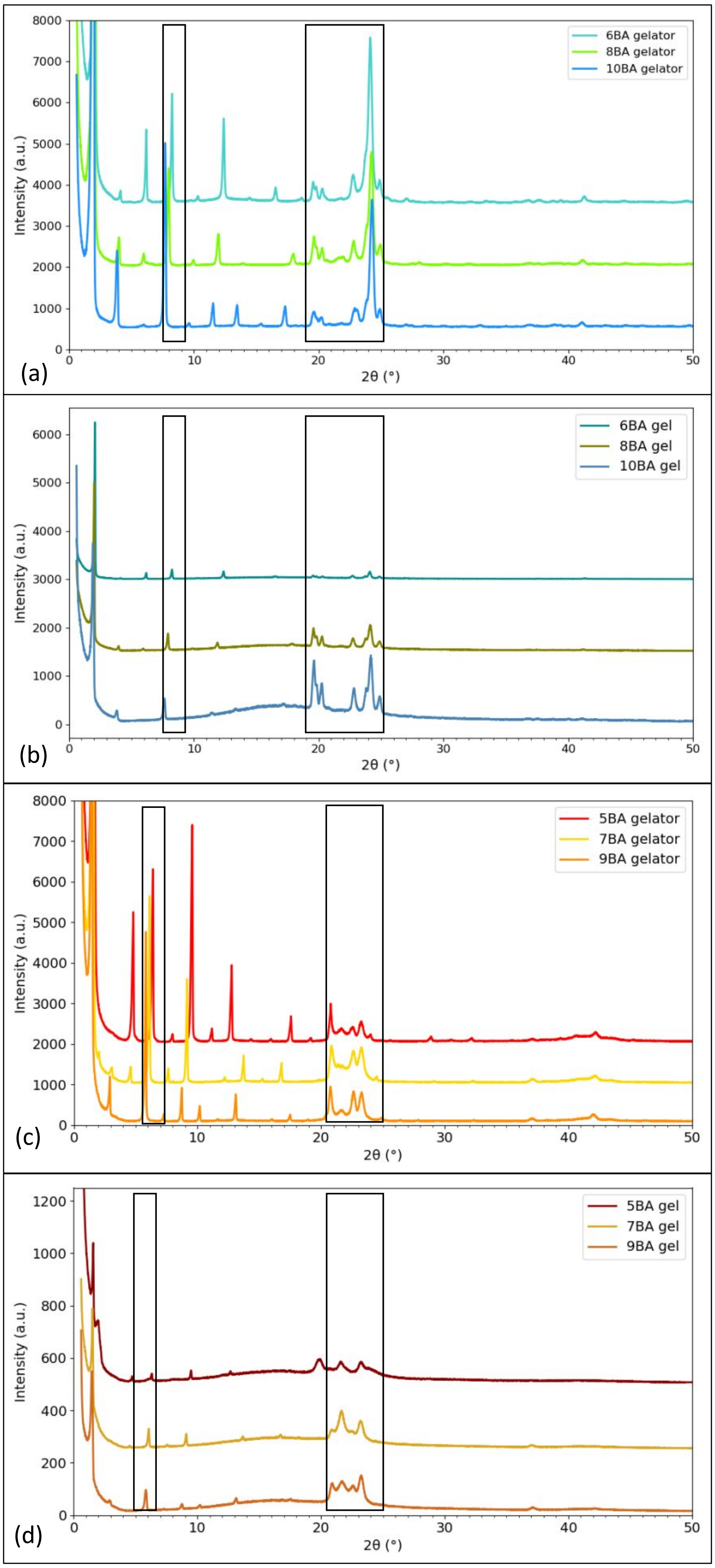

Figure S7. Observed XRD patterns of nBA gelators and gels, a) even nBA gelators in the solid state, b) even nBA gels (20 wt%), c) odd nBA gelators in the solid state, d) odd nBA gels (20 wt%), curves were normalised to the highest intensity and shifted vertically for clarity (the boxes have been added to

guide the eyes toward the similarities and differences between the characteristic reflections in the patterns of the solid and gel states).
